# Personalizing Nutritional Therapy in Pediatric Oncology: The Role of Gut Microbiome Profiling and Metabolomics in Mitigating Mucositis and Enhancing Immune Response to Chemotherapy

**DOI:** 10.3390/children13020293

**Published:** 2026-02-20

**Authors:** Piotr Pawłowski, Natalia Zaj, Kamil Iwaniszczuk, Izabela Grzelka, Wojciech Makuch, Emilia Samardakiewicz-Kirol, Aneta Kościołek, Marzena Samardakiewicz

**Affiliations:** 1 Department of Psychology, Psychosocial Aspects of Medicine, Faculty of Medicine, Medical University of Lublin, 20-093 Lublin, Poland; 2 Institute of Medical Sciences, University of Applied Sciences in Chełm, 22-100 Chełm, Poland; 3 Department of Emergency Medicine, University Children’s Hospital in Lublin, 20-093 Lublin, Poland; 4 Puławy Hospital—Independent Public Health Care Facility, 24-100 Puławy, Poland; 5 Simulation Laboratory for Patient Safety, Department of Medical Education, Medical University of Lublin, 20-093 Lublin, Poland; 6 Department of Nursing Development, Faculty of Health Sciences, Medical University of Lublin, 20-081 Lublin, Poland

**Keywords:** dietary interventions, gut microbiome-targeted therapies, nutrition supplements, pediatric oncology

## Abstract

Introduction: Intensive chemotherapy protocols and hematopoietic stem cell transplantation (HSCT) in children with cancer frequently lead to severe complications, such as mucositis and immune dysfunction. A growing body of evidence indicates that these complications are closely associated with the patient’s nutritional status and the composition of the gut microbiome, which becomes profoundly destabilized as a result of cytotoxic therapy and antibiotic use. Background: The aim of this review is to critically evaluate the current state of knowledge on the interplay between gut dysbiosis, metabolomic profiles—with particular emphasis on short-chain fatty acids (SCFAs)—and treatment-related toxicity in pediatric patients, as well as to delineate pathways toward personalized nutritional therapy. Methods: A narrative review was conducted, including clinical and preclinical studies published between January 2015 and October 2025. PubMed/MEDLINE, Embase, Cochrane Library, and other databases were searched, focusing on changes in microbiome composition, correlations between gut-derived metabolites and the severity of complications (sepsis, graft-versus-host disease [GvHD], mucositis), and the effects of targeted nutritional interventions (probiotics, prebiotics, postbiotics, and fecal microbiota transplantation [FMT]) on microbiome modulation during anticancer therapy. Results: The analysis demonstrates that pediatric oncologic treatment leads to a marked reduction in microbial diversity, including the loss of protective *Clostridiales* taxa (e.g., *Faecalibacterium*), accompanied by an overgrowth of *Proteobacteria* pathobionts. Metabolomic profiling indicates that low SCFA levels (e.g., butyrate < 20–50 µmol/g) are a strong predictor of severe mucositis, prolonged neutropenia, and an increased risk of sepsis. Interventions aimed at restoring eubiosis and enhancing SCFA production show potential in strengthening the intestinal barrier, modulating immune responses, and enabling maintenance of the planned relative dose intensity (RDI) of chemotherapy by reducing treatment-related toxicity. Conclusions: Gut microbiome profiling and fecal metabolomics represent promising prognostic tools in pediatric oncology. There is an urgent need for further research employing “omics”-based approaches to develop precise, individually tailored nutritional protocols. Such strategies, including postbiotics and FMT, may minimize treatment-related adverse effects and improve long-term clinical outcomes in pediatric patients.

## 1. Introduction

Hematological malignancies in children represent a significant challenge for modern medicine. Each year, hundreds of thousands of new cases are diagnosed worldwide, with acute lymphoblastic leukemia (ALL) and acute myeloid leukemia (AML) being the most common entities [1]. While intensive multi-agent chemotherapy protocols, often combined with allogeneic hematopoietic stem cell transplantation (HSCT), have drastically improved survival rates, achieving this cure comes at a high biological cost. The success of these therapies is inextricably linked to the risk of severe, life-threatening complications, as the therapeutic window between eradicating malignant clones and preserving healthy host tissue is notoriously narrow [2,3].

These complications, largely resulting from the systemic toxicity of treatment, primarily include immunosuppression, neutropenia, and damage to rapidly proliferating tissues, particularly the gastrointestinal (GI) epithelium. The pathophysiology of GI toxicity, manifesting clinically as mucositis, involves a complex cascade of oxidative stress, DNA damage, and pro-inflammatory cytokine release that compromises the mucosal barrier. Such toxicity not only significantly reduces the quality of life (QoL) of young patients and necessitates intensive supportive care, but it frequently dictates chemotherapy dose delays or reductions. Suboptimal dosing negatively affects treatment intensity and is directly correlated with higher relapse rates and poorer long-term survival outcomes, including overall survival (OS) [4].

Traditionally, the management of oncological complications has been largely reactive, focusing on supportive care measures such as empirical broad-spectrum antibiotic therapy, opioid analgesics, and parenteral nutrition. However, these interventions often exacerbate underlying GI dysfunction rather than resolving it. In recent years, advances in high-throughput next-generation sequencing (NGS) technologies, including 16S rRNA gene sequencing and whole-genome shotgun metagenomics, have shifted research attention toward the integral role of the gut microbiome. These tools have revealed that the gut is not merely a passive victim of chemotherapy but an active participant in modulating systemic drug toxicity and immune response [5,6].

Gut dysbiosis is defined as a qualitative and quantitative imbalance in the ecosystem of microorganisms inhabiting the gastrointestinal tract. In children undergoing chemotherapy and HSCT, dysbiosis is common and is characterized by a dramatic reduction in microbial diversity. A depletion of protective, anti-inflammatory taxa, such as Clostridiales (e.g., *Faecalibacterium prausnitzii* and *Roseburia*), is observed concurrently with an overgrowth of opportunistic pathobionts, including Proteobacteria and Enterococcus. These alterations are induced by a multi-hit mechanism: prophylactic and therapeutic antibiotic use eradicates commensal flora, altered dietary intake deprives microbes of essential substrates, and cytotoxic agents directly damage bacterial cell walls [7,8]. A key mechanistic link between this dysbiosis and clinical complications is the perturbation of the fecal metabolome, particularly the depletion of bacteria-derived metabolites known as short-chain fatty acids (SCFAs) chiefly butyrate, acetate, and propionate. SCFAs serve critical physiological functions as the primary energy source for colonocytes, enhancers of epithelial tight junctions, and potent epigenetic regulators of mucosal immunity [9,10].

A growing body of clinical and preclinical evidence indicates a robust causal relationship between the severity of dysbiosis, the subsequent reduction in SCFA production, and the incidence of major clinical morbidities. Depleted butyrate levels impair mucosal healing, thereby intensifying mucositis. Furthermore, the loss of barrier integrity allows for bacterial translocation, increasing the risk of bloodstream infections and sepsis. In the context of HSCT, the absence of SCFA-mediated regulatory T cell (Treg) induction exacerbates alloreactivity, significantly heightening the risk of graft-versus-host disease (GvHD). Understanding this microbiota-metabolite-immune axis opens new avenues for proactive therapeutic interventions that extend beyond the current standard, mostly generic guidelines of the North American Society for Pediatric Gastroenterology, Hepatology and Nutrition (NASPGHAN) and the European Society for Clinical Nutrition and Metabolism (ESPEN) [11,12,13,14,15].

Despite the growing recognition of the microbiome’s importance, a significant literature gap remains. While numerous studies describe general dysbiosis in adult oncology patients, there is a paucity of comprehensive reviews integrating the crucial role of the fecal metabolome, specifically SCFA depletion, with the clinical cascade of mucositis, sepsis, and GvHD in the uniquely vulnerable pediatric population. Furthermore, the current literature often treats the microbiome merely as a biomarker of injury, lacking a synthesis that translates these multi-omics findings into actionable, personalized nutritional protocols. There is an unmet need to bridge the scattered multidimensional data (microbiomics, metabolomics, and clinical outcomes) to provide a foundation for future precision interventions.

The aim of this review is to critically evaluate and synthesize current knowledge on the interrelationships between gut dysbiosis and metabolomic profiles, with particular emphasis on SCFA and treatment-related toxicity, including mucositis, sepsis, and GvHD in pediatric patients treated for hematological and oncological malignancies. The review provides added value through the integrated linkage of three key components: microbiomics, metabolomics, and clinical correlations.

The paper focuses on the concept of personalized nutritional therapy (Precision Nutritional Therapy). It substantiates the need to employ fecal metabolomic profiling as a tool for individualized intervention strategies aimed at selectively increasing the production of butyrate and other SCFAs. This represents a step toward the development of protocols designed to optimize the gut–immune axis in order to minimize treatment-related toxicity and improve the long-term effectiveness of oncological therapies in the youngest patients.

## 2. Materials and Methods

### 2.1. Literature Search Strategy

This review was conducted in accordance with evidence synthesis methodology and the principles of narrative review reporting. A comprehensive search of both the clinical and preclinical literature was performed using the following scientific databases: PubMed/MEDLINE, Embase, the Cochrane Library, Web of Science, and Google Scholar.

#### 2.1.1. Time Frame and Language Criteria

The search covered publications published between January 2015 and October 2025. Articles published in English and Polish were included.

#### 2.1.2. Keywords and Search Query Construction

The search strategy was based on a combination of controlled vocabulary terms from thesauri (Medical Subject Headings, MeSH) and free-text keywords, linked using logical operators AND and OR.

The main keyword categories were structured according to the PICA framework (Population, Intervention/Exposure, Comparator, Outcome):

1. Population: Pediatric OR children OR adolescent OR oncology OR leukemia OR acute lymphoblastic leukemia (ALL) OR acute myeloid leukemia (AML) OR hematopoietic stem cell transplantation (HSCT).
2. Exposure (Microbiome/Metabolome): Gut microbiome OR microbiota OR dysbiosis OR metabolomics OR short-chain fatty acids (SCFAs) OR butyrate OR propionate OR acetate.
3. Outcome (Toxicity/Effect): Mucositis OR sepsis OR intestinal inflammation OR gastrointestinal toxicity OR graft-versus-host disease (GvHD) OR immune function OR T cell OR probiotic OR prebiotic OR synbiotic OR postbiotic OR nutritional intervention.

An example search query (adapted for each database) was as follows: (Pediatric OR children) AND (gut microbiome OR dysbiosis) AND (short-chain fatty acids OR butyrate) AND (mucositis OR toxicity).

### 2.2. Inclusion and Exclusion Criteria

#### 2.2.1. Inclusion Criteria

The following types of studies were included in the analysis:

- Clinical Studies—randomized controlled trials (RCTs), prospective and retrospective cohort studies, and pilot studies.
- Preclinical/Experimental Studies—studies conducted in animal models (primarily murine models of mucositis or graft-versus-host disease) providing mechanistic evidence (for example, the role of short-chain fatty acids in immunomodulation and intestinal barrier integrity).
- Review Articles and Meta-Analyses—included as reference sources to identify additional key publications using a snowballing approach.
- Publications focusing on:
  · Correlations between changes in microbiome composition (16S ribosomal ribonucleic acid sequencing or shotgun metagenomic sequencing) and clinical outcomes.
  · Associations between metabolites (measurement of short-chain fatty acid concentrations in stool or serum) and the severity of complications.
  · The impact of nutritional interventions on microbiome modulation in pediatric patients undergoing oncological treatment.

#### 2.2.2. Exclusion Criteria

The following were excluded from the analysis:

- Case reports and letters to the editor.
- Studies focusing exclusively on adult populations (>18 years of age).
- Publications not classified as peer-reviewed scientific articles (for example, conference abstracts without full-text availability).
- Articles that did not provide quantitative microbiome or metabolomic data.

### 2.3. Study Selection and Data Extraction Process

#### 2.3.1. Study Selection

The selection process was conducted in two stages and performed by two independent reviewers (within the context of the project):

1. Titles and abstracts were screened to exclude irrelevant publications or those not meeting the inclusion criteria.
2. Full texts of potentially relevant articles were assessed to confirm final eligibility. Discrepancies between reviewers were resolved through discussion or consultation with a third independent researcher.

#### 2.3.2. Data Extraction

From each included study, the following key information was extracted and compiled into a structured virtual table:

- Study Identification—authors, year of publication, study design, population characteristics (number of patients, age, type of malignancy and treatment).
- Microbiome Analysis Methods—analytical method and taxonomic resolution.
- Metabolomic Analysis Methods—analytical technique and concentrations of key short-chain fatty acids.
- Clinical Outcomes—severity of mucositis (according to the World Health Organization scale or the Oral Mucositis Assessment Scale), incidence and type of infections (sepsis), and risk of graft-versus-host disease.
- Type of Intervention—probiotic, prebiotic, dietary intervention, or combined approaches.
- Key Findings—reported correlations between microbiome composition or short-chain fatty acid levels and treatment-related toxicity or intervention effectiveness.

### 2.4. Quality Assessment

Included clinical and cohort studies were assessed for the risk of bias using standardized tools, such as the Methodological Index for Non-Randomized Studies (MINORS) for non-randomized studies, and the Cochrane Risk of Bias Tool version 2.0 (RoB 2.0) for randomized controlled trials. Only studies of high or moderate methodological quality were included in the narrative synthesis.

### 2.5. Data Synthesis Methods

Data were synthesized using a narrative synthesis approach. A formal statistical meta-analysis was not performed due to substantial heterogeneity in chemotherapy protocols, types of nutritional interventions, microbiome sequencing techniques, and metabolite measurement methods.

The narrative synthesis focused on:

1. Identification of consistent and reproducible dysbiosis patterns associated with clinical complications.
2. Determination of short-chain fatty acid thresholds associated with increased risk.
3. Integration of mechanistic (preclinical) and clinical evidence regarding the impact of interventions on the microbiome–metabolome–immune axis.

The results are presented in clear thematic subsections aligned with the objectives of the review.

## 3. Results

### 3.1. Gut Microbiome Profiling and Fecal Metabolomics as Predictors of Toxicity

#### 3.1.1. Intestinal Dysbiosis in Pediatric Patients Undergoing Chemotherapy and HSCT

Intensive chemotherapy regimens and HSCT in pediatric patients with hematological malignancies, such as ALL and acute myeloid leukemia (AML), profoundly disrupt the gut microbiome. These interventions result in a marked reduction in overall microbial diversity, as assessed by alpha-diversity metrics including observed operational taxonomic units (OTUs), the Shannon index, and Faith’s Phylogenetic Diversity [5]. This decline typically occurs rapidly after treatment initiation, with studies reporting a 50–80% loss in microbial richness and evenness within 7–28 days of induction chemotherapy or conditioning regimens. Reduced diversity often persists throughout periods of neutropenia and engraftment, with only partial recovery observed at later follow-up time points [16]. The combined effects of cytotoxic agents, broad-spectrum antibiotics (e.g., meropenem), and immunosuppressive therapies further exacerbate dysbiosis, leading to ecosystem instability and increased susceptibility to complications such as infections, enterocolitis, and GvHD [17]. Notably, pre-treatment microbiome profiles in pediatric patients already demonstrate reduced baseline diversity compared with age-matched healthy controls. Subsequent deterioration during therapy underscores the particular vulnerability of the immature pediatric gut ecosystem [18]. Key taxonomic alterations in the gut microbiome of pediatric patients undergoing chemotherapy and HSCT include a significant reduction in the relative abundance of the Firmicutes and Bacteroidetes phyla. These taxa typically dominate the healthy pediatric gut and play essential roles in metabolic stability, anti-inflammatory signaling, and SCFA production [16]. This depletion is particularly evident following induction chemotherapy. Although Firmicutes often remain the predominant phylum, there is a qualitative decline in beneficial genera such as Blautia and Faecalibacterium. In parallel, marked reductions in Bacteroidetes contribute to ecosystem instability and impaired mucosal barrier function [19]. Concurrently, a pronounced expansion of Proteobacteria, especially members of the Enterobacteriaceae family (e.g., Klebsiella and Escherichia), emerges as a hallmark of dysbiosis and microbial instability. This shift correlates with an increased risk of bacterial translocation, systemic inflammation, and infectious complications in patients with hematological malignancies [20]. These alterations are further intensified by conditioning regimens and antibiotic prophylaxis, which promote pathobiont overgrowth and reduce microbial resilience. Longitudinal 16S rRNA sequencing studies consistently demonstrate Proteobacteria dominance during neutropenic phases [21]. In pediatric cohorts with AML, such phylum-level shifts are associated with prolonged neutropenia and poorer clinical outcomes, highlighting the prognostic relevance of microbiome monitoring [16].

#### 3.1.2. Depletion of Protective Taxa and Expansion of Pathobionts

Protective taxa within the pediatric gut microbiome, particularly butyrate-producing members of the Clostridiales order, such as *Ruminococcaceae* (e.g., *Faecalibacterium prausnitzii*, *Ruminococcus*, and *Subdoligranulum*), play a central role in maintaining intestinal barrier integrity and immune homeostasis. These effects are mediated through the production of SCFAs, including butyrate, which supports epithelial cell metabolism, enhances tight junction integrity, and modulates anti-inflammatory responses via regulatory T-cell induction [17]. These commensal taxa are frequently depleted following chemotherapy and HSCT, resulting in reduced SCFA availability and increased susceptibility to complications such as acute GvHD and systemic inflammation, as demonstrated in longitudinal studies of pediatric leukemia patients [20]. In contrast, pathobionts, including opportunistic bacteria from the Enterococcaceae and Enterobacteriaceae families (e.g., *Enterococcus* spp. and *Klebsiella*), expand under dysbiotic conditions. Their proliferation has been linked to sepsis through enhanced biofilm formation, antibiotic resistance, and bacterial translocation, particularly in immunocompromised children undergoing induction chemotherapy for AML [17]. This dichotomy, characterized by the loss of protective butyrate-producing commensals alongside the expansion of sepsis-associated pathobionts, underscores the prognostic significance of microbiome profiling. Higher pre-HSCT diversity of beneficial taxa has been associated with improved survival and reduced infection rates in pediatric cohorts [20]. Moreover, therapeutic strategies aimed at restoring protective microbial communities, such as fecal microbiota transplantation (FMT), have shown promise in reducing sepsis risk by re-establishing butyrate-producing taxa and suppressing pathobiont dominance in neonatal and pediatric bacterial infections [20].

### 3.2. Metabolomic Correlates of Treatment Toxicity: The Role of SCFAs

Decreased fecal concentrations of SCFAs, including butyrate, acetate, and propionate, have been identified as predictive biomarkers of heightened treatment-related toxicity in pediatric patients undergoing chemotherapy and HSCT. Furthermore, quantitative analyses reveal significant correlations between these low SCFA levels and an increased severity of complications, such as neutropenia, mucositis, and GvHD [18].

Longitudinal metabolomic profiling in pediatric ALL cohorts demonstrates that baseline butyrate levels below 20–50 μmol/g post-induction chemotherapy are associated with prolonged neutropenia episodes exceeding 14 days and elevated infection risks. Conversely, higher pre-treatment concentrations (>80 μmol/g) correlate with complication rates that are reduced by 30–40% [5]. Similarly, in allogeneic HSCT recipients, reduced acetate and propionate levels (typically <40 μmol/g during engraftment) predict acute GvHD onset with odds ratios of 2.5–3.0. This prediction is supported by gas chromatography-mass spectrometry data, which links these SCFA deficits to impaired epithelial barrier integrity and systemic inflammation [17]. These quantitative thresholds underscore the prognostic utility of SCFA monitoring. Specifically, declines in total SCFA pools (from baseline means of 100–150 μmol/g to <50 μmol/g peri-transplant) are independently associated with sepsis incidence rates surpassing 25% and delayed hematopoietic recovery. Ultimately, this robust association highlights dysbiosis-driven metabolic perturbations as key risk factors in pediatric hematological malignancies [20]. Preclinical and clinical evidence from 2025 studies further confirms that restoring SCFA levels through microbiota modulation could mitigate these risks. Specifically, low butyrate correlates with elevated proinflammatory cytokine profiles (e.g., IL–6 > 100 pg/mL) and mucositis grades ≥3 in over 50% of affected cases [18].

Butyrate serves as the principal energy substrate for colonocytes, accounting for approximately 70% of their metabolic fuel requirements. This energy provision facilitates ATP production via beta-oxidation in the mitochondria. Consequently, it supports epithelial cell proliferation and differentiation, which is crucial for maintaining intestinal barrier integrity during chemotherapy-induced damage [22].

In pediatric HSCT and chemotherapy contexts, butyrate enhances the expression of tight junction proteins, such as occludin, claudin–1, and zonula occludens–1 (ZO–1). This upregulation reduces paracellular permeability and actively prevents bacterial translocation. These protective effects are evident in preclinical models of mucositis, where butyrate supplementation successfully restores barrier function and mitigates histological scores of epithelial injury by 40–60% [22].

**Figure 1:**
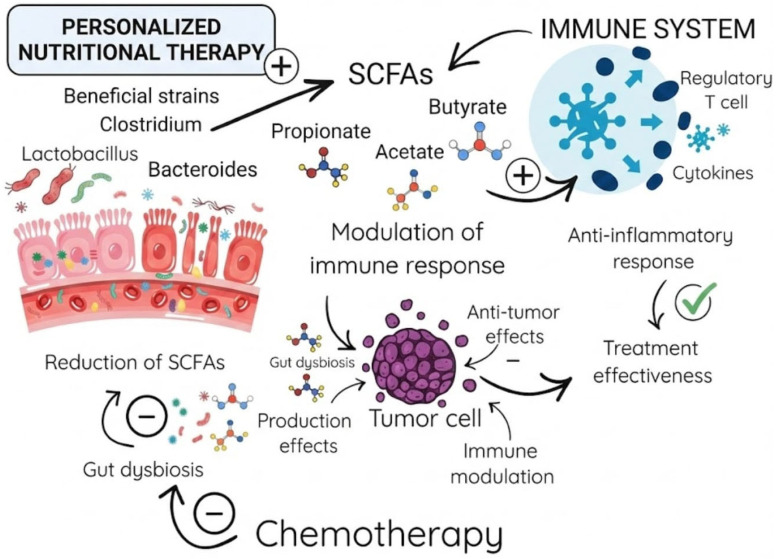
The Gut Microbiota–SCFA–Immune System Axis in Cancer Therapy.

Furthermore, butyrate modulates local inflammatory responses through histone deacetylase (HDAC) inhibition. This crucial action suppresses NF–κB signaling and the subsequent release of proinflammatory cytokines, including TNF–α, IL–6, and IL–1β. Simultaneously, butyrate promotes anti-inflammatory pathways via FOXP3 upregulation and regulatory T-cell (Treg) differentiation. Ultimately, these combined mechanisms lead to significantly reduced neutrophil infiltration and lower oxidative stress in mucosal tissues. Clinical evidence from 2025 pediatric studies indicates that low butyrate levels (<30 μmol/g) correlate with severe mucositis (grades 3–4). Conversely, therapeutic butyrate interventions decrease inflammation markers (e.g., calprotectin) by up to 50% and accelerate mucosal healing, highlighting its dual role in energy provision and immunomodulation. These mechanisms are particularly relevant in pediatric oncology, where butyrate’s anti-apoptotic effects via GPR109A receptor activation further protect against chemotherapy-induced apoptosis. Consequently, this protective pathway offers a strong rationale for developing metabolomic-guided therapies to alleviate mucositis severity [22]. The association between SCFAs and the immune response, as well as their impact on chemotherapy, is presented in Figure 1.

### 3.3. Potential Nutritional Therapy for Gut Microbiome Modulation

#### 3.3.1. Probiotics

Probiotics are microorganisms that offer multiple health benefits when consumed in adequate doses [23]. They produce antimicrobial substances, inhibit the production of toxins, improve mucosal IgA production, modulate immunity, and compete with pathogenic bacteria for binding to the epithelial surface [24,25]. In acute leukemia, probiotics may be beneficial in reducing the risk of infection, promoting the proper function of the gut, and improving intestinal recovery after treatment-related damage [26]. In a randomized, single-blind, pilot study performed by Reyna-Figueroa et al., the authors studied the effect of probiotic supplementation on gastrointestinal negative side effects of chemotherapy. They included 60 patients with acute leukemia, each one after 30 days of intensive chemotherapy. The patients were equally divided into two groups. The first group received *Lactobacillus rhamnosus* GG probiotic supplementation (they administered 5 × 109 CFU per sachet, twice daily, per os), and the second one was with no probiotic supplementation. In the non-supplementation group, 63% of children experienced chemotherapy-related negative side effects, including nausea, vomiting, abdominal distension and diarrhea, compared with 30% of patients in the supplementation group, who reported GI (gastrointestinal) adverse effects (*p* < 0.05) [27]. Moreover, studies have shown that *Lactobacillus rhamnosus* reduces the adverse effects of chemotherapy by modulating the immune system [28]. Furthermore, probiotics containing *Bifidobacterium* strains have been shown to reduce chemotherapy-associated mucositis and diarrhea related to radiotherapy [29].

Despite the favorable outcomes in leukemia treatment, a subset of patients still requires HSCT. Some studies associate complications such as GvHD onset with the changes in the intestinal microbiota [26]. In patients with GvHD, the gut microbiota diversity is observed, as well as a predominance of pathogenic strains. Probiotic supplementation may be beneficial in restoring the intestinal microbiota [15]. A clinical trial conducted by Ladas et al., investigated the feasibility and safety of probiotics in children and adolescents undergoing HSCT. The patients between the ages of 2 and 17 years were included in the study. The patients received the oral probiotic containing *Lactobacillus plantarum* (LBP) beginning on day −8 or −7, continued through day +14 post-transplant. A daily administration of LBP was safe and feasible in patients undergoing HCT. Additionally, no adverse events related to the use of probiotics were observed [30]. Diversity in the composition of the intestinal microbiota is being investigated in human and in cell lines. Simms-Waldrip et al. observed a decrease in the number of *Clostridia* and a rise in the number of *Enterobacteriaceae* in children who developed GvHD [31]. Moreover, treatment with *Clostridia* strains producing high levels of butyrate mitigates acute GvHD [32]. A randomized phase III trial investigates the effect of LBP in preventing acute GvHD in children after allo-HSCT (NCT03057054). The safety of administered per os LBP strains 299 and 299v are being evaluated based on the incidence of LBP bacteremia [33]. Besides the many positive aspects of the use of probiotics, the risk of developing bacteremia still constitutes a potential challenge in hematooncology patients [34,35]. Another factor favoring probiotics is their anticancer potential in in vitro studies observed on cancer cell lines. Mangrolia et al., performed a study on the MCF-7 cell line described antibacterial and anticancer activity of *Staphylococcus xylosus* strains [36]. In the study of Tarrah et al., the results revealed that two strains of *Streptococcus thermophilus* showed stimulation of folate production and anticancer activity. Potential characteristics of M17PTZA496 and TH982 strains, in some patients, seem to be superior to the popular probiotic strain *Lactobacillus rhamnosus* [37].

#### 3.3.2. Prebiotics

Prebiotics are dietary components that feed beneficial intestinal bacteria by fermentation. Gut bacteria break down prebiotics, producing SCFAs and favoring the growth of *Bifidobacterium* and *Lactobacillus* strains [38]. Additionally, these nondigestible fibers enhance chemotherapy effectiveness by overcoming drug resistance and decreasing treatment-related toxicity [39,40,41]. The most common prebiotics include inulin, fructooligosaccharide (FOS), resistant starches and galactooligosaccharides (GOS) [25,42]. They are found in certain foods, such as onion, asparagus, garlic, oats and human milk. In a pilot study conducted by Khandelwal et al., the effect of human milk administration on gut inflammation was evaluated in children after HSCT. The study included patients aged 0–5 years, of whom 32 received human milk (breastfeeding babies *n*  =  9, donor milk *n*  =  23) and 14 received a standard formula supervised by a dietitian. Research has shown that human milk feeding lowers the prevalence of pathogenic species in stool samples as well as decreases markers of gut inflammation [43]. A recent study, conducted by Ren et al., showed that a soy–whey mixture significantly improved muscle condition in acute leukemia survivors. These results suggest an important role of the intestinal microbiota in regulating muscle metabolism [44]. In another study of the addition of a soy–whey mixture was conducted in an Allogeneic Transplanted Mice. The results revealed that the addition of a protein mixture improved the reconstitution of the immune system and prompted the restoration of hematopoietic functions [45]. In studies conducted by Bindels et al., in murine leukemia models, increased levels of butyrate and propionate were observed following the inulin supplementation. A reduction in liver metastases was also reported [46,47]. Moreover, pectic oligosaccharides supplementation was shown to reduce adipose tissue loss and delay the onset of anorexia associated with cancer progression better compared to the inulin implementation [46]. There are two clinical trials, the first one investigates the effect of the inulin administration vs. placebo on pediatric patients undergoing HSCT in the United States (NCT04111471), and the second one, a single-center prospective study regarding 2′-fucosyllactose (2FL) supplementation in pediatric allo-HSCT recipients (NCT04263597) [48,49]. Both have recently completed and the results are pending. The comparison of above-mentioned trials is placed in the table below (Table 1).

#### 3.3.3. Postbiotic

Postbiotics, according to a new definition introduced in 2021, are a “preparation of inanimate microorganisms and/or their components that confers a health benefit on the host” [50]. These molecules or products from probiotic bacteria support the maintenance of intestinal homeostasis [15]. Major products of prebiotic metabolism are butyrate and related SCFA [51]. The use of postbiotics does not increase the risk of bacteremia, because there is a lack of microbe-associated molecular patterns that could potentially induce inflammation and activate innate immune mechanisms, compared to live microorganisms [52]. Chuah et al. studied the cytotoxicity effects of postbiotic metabolites on various cancer cell lines. The researchers evaluated metabolites produced by six strains of LBP and demonstrated selective cytotoxicity via induction of apoptosis and antiproliferative effect in cancer cells, with preservation of healthy cells [53]. Pulliam et al. described the effect of butyrate on 2-fold caspase-3 induction and a 60% reduction in cell viability in various human acute leukemia cells. Within 24 h, butyrate reduced the levels of CCL5 and CCL2 chemokines in U937 and HL-60 cells as well as decreased the levels of CCL5 in THP-1 leukemia cells. In addition to promoting apoptosis, butyrate has the potential to modulate the tumor microenvironment through the induction of differential cytokine expression [54]. Bryndza sheep cheese contains lactic acid bacteria (LAB), with many probiotic effects such as immunomodulatory effects, bacteriocin production and pathogen inhibition [55]. Esmail et al. analyzed the effects of Bryndza sheep cheese powder on SCFA production and gut microbiota. Researchers studied an in vitro model using fecal samples collected from pediatric patients with ALL. Following incubation, increased SCFA production and an enrichment of SCFA-producing bacteria were observed, indicating that dietary intervention may modulate gut microbiota composition and metabolite profiles [56]. Another objective of prebiotic use is the reduction in bloodstream infection (BSI), which is a common complication of leukemia treatment. In a study conducted by Song et al., the changes in the fecal and gut microbiome were investigated using a pediatric T-cell acute lymphocytic leukemia (T-ALL) mouse model. Researchers demonstrated a reduction in SCFA concentration and decreased butyrate-producing *Clostridia* spp. in the model during BSI. Interestingly, SCFA supplementation was observed to attenuate the development of BSI in ALL [57]. A study on 20 children undergoing HSCT examined the role of parenteral nutrition vs. enteral nutrition on SCFA production. A total of 104 fecal samples were analyzed. Samples were taken before transplant and at multiple time points following HSCT, up to 120 days post-transplantation. Enteral nutrition promoted the restoration of more beneficial intestinal microbiome, caused faster amelioration of SCFA production and decreased the risk of treatment-related complications, including infections and GvHD [58]. In another study, enteral nutrition was shown to have a significant role in reducing the risk of acute GvHD, especially the intestinal acute GvHD and grades III-IV. Therefore, early implementation of parenteral nutrition as first-line nutritional support seems to represent an important element of the therapeutic approach in patients undergoing HSCT [59].

#### 3.3.4. Fecal Microbiome Transplantation

FMT is a microbiome-based therapy that refers to the implementation of fecal samples from healthy donors to a recipient with dysbiotic gut microbiota, aiming to improve and restore intestinal homeostasis [60]. To minimize the risk of potential infection in immunocompromised patients, rigorous sterility methods and extensive donor screening are implemented prior to administration of donor fecal suspension [61]. The insertion of FMT includes nasogastric tube injection, oral delivery, enema or colonoscopy [5]. Due to the modulation of the gut microbiota composition in the host, FMT is regarded as the “ultimate probiotics” [62]. In children with leukemia, FMT represents a promising therapeutic option for patients colonized with multidrug-resistant bacteria, in the management of steroid-refractory GvHD as well as in therapy of recurrent *Clostridioides difficile* infections [63,64,65]. Table 2 summarizes the studies regarding FMT implementation in HSCT patients, mainly in the pediatric population. Current studies indicate that the efficacy of FMT exceeds that of single-strain therapies, due to its ability to restore the overall microbial community [66,67]. Although the available research suggests several potential benefits, they are predominantly based on small cohorts and often limited to pediatric case reports [68]. Therefore, larger, prospective studies especially on the pediatric population are needed to accurately assess safety and to support the development of standardized FMT protocols, which may be essential for the future development of this microbiota-modulating approach [5,69].

### 3.4. Improvement in Long-Term Clinical Outcomes and QoL

#### 3.4.1. Correlation Between Microbiome Modulation and Chemotherapy Dose Intensity

Interactions between the gut microbiota and the host organism play a key role in regulating intestinal homeostasis and modulating the inflammatory response. Relative dose intensity (RDI) describes how much of the planned dose of cytostatic drugs a patient actually receives per unit of time. Severe adverse events such as diarrhea, mucositis, neutropenic fever, and prolonged neutropenia are the most common causes of treatment cycle delays and dose reductions, which directly reduce RDI. For this reason, maintaining intestinal eubiosis by reducing toxicity and infectious complications may increase the likelihood of completing treatment as planned and maintaining the intended dose intensity [76,77].

Commensal gut microbiota participates in maintaining the integrity of the intestinal barrier by reducing epithelial permeability, limiting enterocyte apoptosis, and strengthening tight junctions. The stability of the gut microbiome may promote the maintenance of full chemotherapy dosage intensity [78]. Monitoring and modulation of the gut microbiota may be a potential tool for predicting response to anticancer treatment and may enable more effective management of gastrointestinal and systemic complications associated with immunosuppression [79].

Research by Ling Wei and co-authors on chemotherapy-induced intestinal mucosal damage (IMD) indicates that this process is primarily characterized by a decrease in the diversity and total number of intestinal bacteria, a reduction in the number of Gram-positive bacteria, a relative increase in Gram-negative bacteria, and a decrease in the number of commensal microorganisms with a simultaneous increase in potentially pathogenic bacteria. It has also been shown that chemotherapy-induced IMD negatively affects intestinal stem cells and disrupts the homeostasis of the intestinal epithelium. In addition, chemotherapy leads to changes in the expression of Toll-like receptor (TLR) signaling pathways, especially TLR2 and TLR4, resulting in increased expression of pro-inflammatory mediators within the intestinal mucosa. These data indicate that chemotherapy induces IMD, and interactions between the intestinal microbiota and TLR may further exacerbate the local inflammatory response [80]. Recent studies indicate that modulation of the gut microbiota is a promising strategy for reducing the toxicity of combination therapies without significantly reducing their anticancer efficacy [81].

The variation in gut microbiota composition and β-glucuronidase bacterial activity between individual patients is of significant clinical importance, which partly explains the observed differences in the toxicity and therapeutic efficacy of irinotecan. This approach may facilitate the optimization of dosing regimens, better toxicity management, and maintenance of the anticancer activity of treatment [82,83].

A particularly important example of microbiome intervention in the pediatric population is the study by Furman et al., which showed that modulation of the gut microbiota through the administration of cefixime allowed for the escalation of irinotecan doses in children. The combination therapy was well tolerated and allowed for an increase in the dose of the drug in patients for whom diarrhea had previously been a limiting factor in treatment. This study is one of the few examples in which an intervention targeting the gut microbiota directly translates into a dosage parameter, i.e., a higher tolerated dose and greater drug exposure [84].

A study by Hakim et al. conducted in a cohort of 199 children with newly diagnosed ALL showed that the dominance of *Enterococcaceae* or *Streptococcaceae* taxa was associated with a higher risk of subsequent episodes of neutropenic fever and diarrhea, which are common causes of hospitalization, delays in treatment cycles, and modifications to chemotherapy doses [85].

Delayed neutrophil recovery during treatment of ALL increases the risk of infection and leads to delays in chemotherapy. Emerging evidence points to the involvement of the gut microbiota in neutrophil reconstitution after chemotherapy. Sørum and co-authors investigated the interactions between the gut microbiota and neutrophil dynamics in 51 children with newly diagnosed ALL. The analysis included daily measurements of absolute neutrophil count (ANC), weekly measurements of chemokine concentrations (CXCL1, CXCL8) and granulocyte colony-stimulating factor (G-CSF), as well as 16S rRNA sequencing of the gut microbiota. A significant reduction in bacterial diversity and an excessive increase in the *Enterococcus* genus were demonstrated during induction therapy. Prolonged neutropenia was associated with a reduced abundance of commensals from the *Ruminococcaceae* and *Lachnospiraceae* families and an excessive growth of *Enterococcus*, indicating that dysbiosis may contribute to delayed neutrophil reconstitution and an increased risk of infectious complications [18,86].

Longitudinal studies conducted in children with ALL analyzed changes in the gut microbiota at successive stages of treatment from diagnosis, through induction and consolidation, to maintenance therapy. Assessment of alpha diversity using the Shannon index, based on OTU—97% sequence similarity, showed a significant reorganization of the gut microbiome during therapy. The early stages of chemotherapy were not associated with a significant increase in diversity, but a statistically significant change in the composition of the microbiota was observed between the beginning and end of treatment. However, it should be emphasized that an increase in microbial diversity is not synonymous with a full restoration of eubiosis or functional improvement of the host’s intestinal ecosystem [87,88].

#### 3.4.2. Reduction in Long-Term Complication Risk: Impact on Chronic Intestinal Inflammation and the Risk of Secondary Cancers

In pediatric patients with leukemia, there are clear associations between reduced gut microbiota diversity and severe treatment complications, including infections, increased risk of GvHD, and increased mortality after allo-HSCT. These observations emphasize that long-term microbial dysbiosis is not merely a transient effect of treatment but may play an important role in the pathogenesis of late health effects after cancer therapy in children [89].

In the context of secondary gastrointestinal cancers, the genotoxic potential of selected intestinal pathobionts is emphasized. Escherichia coli strains with the pks pathogenicity island produce colibactin, which induces DNA (deoxyribonucleic acid) damage and leaves a characteristic mutational signature detected in intestinal cancer tissues. ETBF (enterotoxigenic *Bacteroides fragilis* strains), through the BFT (fragilisin) toxin, can also initiate pro-inflammatory and pro-carcinogenic cascades and promote epithelial damage and dysplastic processes. In the pediatric population, clinical evidence is still limited, but persistent dysbiosis after treatment is a biologically plausible mechanism modifying the risk of late complications, including carcinogenesis [77,90,91].

In addition, there are reports that persistent changes in the gut microbiota correlate not only with complications during treatment but also with subsequent immune, metabolic, and psychological disorders that affect the QoL of cancer survivors. The Chemo-Gut study protocol indicates that chemotherapy can cause long-term dysbiosis, increased intestinal permeability, and chronic inflammation, which may be associated with long-term health consequences such as metabolic disorders, immune changes, and reduced QoL after treatment [88]. A study by Chua and co-authors revealed a correlation between dysbiosis and increased activation of T lymphocytes and certain biomarkers of inflammation, namely circulating interleukin IL-6 and C-reactive protein (CRP) levels. The study was conducted in adult patients who had survived childhood [92].

Adult patients who survived ALL were found to have significantly reduced microbial diversity compared to the control group. Reduced microbial diversity is often associated with chronic diseases and chronic inflammation (low-grade inflammation), particularly in the case of secondary cancers, cardiovascular diseases, kidney dysfunction, serious musculoskeletal problems, endocrinopathies, obesity, diabetes, and ischemic heart disease, which are common late effects for CCS (childhood cancer survivors) [77,93]. Among adult survivors of childhood cancer, the prevalence of adverse health outcomes was high, and systematic risk-based medical screening identified a significant number of previously undiagnosed problems that are more common in the older population [94].

From the perspective of the QoL of cancer survivors, it is important to note that persistent dysbiosis may be associated not only with immunological and metabolic parameters but also with chronic symptoms and poorer functioning in the long term. A growing body of evidence points to a link between the composition of the microbiome and the severity of cancer-related fatigue and overall QoL in cancer populations and survivors, making the microbiome a potential target for interventions to support post-treatment rehabilitation [95,96]. For this reason, monitoring the gut microbiota and strategies to support the restoration of eubiosis, such as nutritional interventions or the use of pre- and probiotics within clinical indications and safety guidelines, may be important not only for reducing late somatic complications but also for improving the well-being and comfort of patients after the end of therapy [77].

## 4. Conclusions

Gut microbiome profiling and fecal metabolomic analysis are emerging as key tools for risk stratification of complications in children undergoing intensive chemotherapy and HSCT. Reduced concentrations of SCFAs, particularly butyrate (levels < 20–50 µmol/g), constitute an independent predictive factor for the development of severe mucositis, prolonged neutropenia, and sepsis. Loss of microbial diversity (a decline of 50–80% within 7–28 days from treatment initiation) and specific taxonomic shifts—primarily the depletion of protective bacteria from the order *Clostridiales* (e.g., *Faecalibacterium*, *Blautia*) with concurrent expansion of pathobionts from the phylum *Proteobacteria* (e.g., *Enterobacteriaceae*)—correlate with poorer clinical outcomes and delayed hematologic recovery.

Studies confirm a strong causal relationship between dysbiosis, deficiency of bacterial metabolites, and disruption of the intestinal barrier. Butyrate plays a critical role in maintaining gut barrier integrity by stimulating the expression of tight junction proteins (occludin, claudin-1, ZO-1) and serves as the primary energy source for colonocytes (covering approximately 70% of their energy requirements), thereby counteracting chemotherapy-induced apoptosis. Bacterial metabolites, through inhibition of histone deacetylases (HDACs), suppress pro-inflammatory pathways (NF-κB) and promote the differentiation of regulatory T cells (Tregs), which is crucial for resolving mucosal inflammation and preventing GvHD.

Standard nutritional guidelines (ESPEN, NASPGHAN) require supplementation with microbiota-targeted strategies tailored to individual patient profiles. Although probiotics may provide some benefit in reducing gastrointestinal symptoms, their use in immunosuppressed patients carries a risk of bacteremia (e.g., caused by *Lactobacillus* strains), limiting their widespread application during acute phases of treatment. Postbiotics (metabolites, non-viable microorganisms) offer a safer alternative, demonstrating selective cytotoxicity against cancer cells while sparing healthy tissues. Prebiotics (e.g., inulin, HMOs) effectively stimulate endogenous SCFA production and may mitigate inflammation. FMT represents a promising approach for “resetting” the gut ecosystem, particularly in the treatment of steroid-refractory GvHD and infections caused by multidrug-resistant organisms; however, protocol standardization in pediatric populations is still required.

The state of the gut microbiome extends beyond nutritional support, directly influencing the efficacy of anticancer therapy. Maintenance of eubiosis and reduction in toxicity (diarrhea, mucositis) enable preservation of the planned chemotherapy dose intensity, which is critical for OS. Microbiota modulation may also permit dose escalation of certain agents (e.g., irinotecan). Persistent dysbiosis in childhood cancer survivors is associated with chronic low-grade inflammation, increased risk of metabolic and cardiovascular diseases, and secondary malignancies (via bacterial genotoxins, such as colibactin).

The analyzed evidence highlights the need to move beyond purely symptomatic management of complications toward active modulation of the gut environment. Implementation of routine fecal metabolomic monitoring and targeted interventions (prebiotics, postbiotics, personalized diets) represents a necessary step toward improving the safety and effectiveness of pediatric oncologic treatment.

## Figures and Tables

**Table 1:** The comparison of clinical trials regarding prebiotic supplementation in pediatric patients undergoing HSCT.

Identification Code	NCT04111471	NCT04263597
Type of trial	randomized	a single center prospective study
The studied substance	inulin	2′-fucosyllactose
Drug administration	orally	orally
Study duration	2019–2024	2020–2025
Clinical trial phase	not applicable	phase I/IIa
Age of the patients	2–18 years	Arm 1: 0–5 years; Arm 2: 5.1–10 years; Arm 3: >10 years
Number of enrolled patients	40	70
Time of administration	from day −7 until day +14 after transplant	from day −7 until day + 30 after HSCT
Organizations involved with this study	Ann & Robert H Lurie Children’s Hospital of Chicago	Children’s Hospital Medical Center, Cincinnati

**Table 2:** The use of FMT in patients suffer from malignant and nonmalignant hematologic conditions requiring HSCT.

Patient Type	Number of Patients	Age Range	Article Type	Main Results	References
refractory acute GvHD ^1^	1	5 years	case study	a clinical symptoms reduction and increased bacterial diversity, an enrichment of *Firmicutes*, and a reduction in *Proteobacteria*	[70]
patients with ALL ^2^ colonized by MDR ^3^ bacteria	5	2–18 years	case series study	80% MDR ^3^ bacteria decolonization within one week	[71]
immunocompromised included: solid organ transplantation malignancy, primary immunodeficiency, or other chronic conditions	59	1.5–18 years	multi-center retrospective cohort study	79% after 1st FMT ^4^, 86% after 1 or more FMT ^4^, no MDR ^3^ infections and no deaths	[72]
children with recurrent CDI ^5^	49	4 to 193 months-old	retrospective study	26.32% short-term negative side effects (mostly mild)	[73]
HSCT ^6^ recipients with CDI ^5^	7	25–67 years	retrospective study	85.7% of FMT ^4^ recipients had no CDI ^5^ recurrence and no serious side effects were identified	[74]
patients with hematologic malignancies (4 after and 6 before allo-HSCT ^6^) colonized with MDR ^3^ bacteria	10	16–64 years	retrospective study	decolonization in 7 of 10 patients, FMT ^4^ was safe: 1 patient had constipation, and 2 had grade I diarrhea	[75]
children with GvHD ^1^ and antibiotic-resistant colitis developed after allo-HSCT ^6^	7	3–10 years	prospective single-centerstudy	6 of 7 patients reached a clinical response, an increased number of *Bacteroides fragilis*, *Escherichia coli* and *Faecalibacterium prausnitzii* in fecal microbiota since day +8 after FMT ^4^ was achieved	[68]

^1^ graft versus host disease (GvHD). ^2^ acute lymphoblastic leukemia (ALL). ^3^ multi-drug resistant (MDR). ^4^ fecal microbiota transplantation (FMT). ^5^ *Clostridium difficile* infection (CDI). ^6^ hematopoietic stem cell transplantation (HSCT).

## Data Availability

Data sharing is not applicable.

## Abbreviations

The following abbreviations are used in this manuscript:

HSCT	Hematopoietic stem cell transplantation
SCFA	Short-chain fatty acid
GvHD	Graft-versus-host disease
FMT	Fecal microbiota transplantation
RDI	Relative dose intensity
ALL	Acute lymphoblastic leukemia
QoL	Quality of life
OS	Overall survival
NASPGHAN	North American Society for Pediatric Gastroenterology, Hepatology and Nutrition
ESPEN	European Society for Clinical Nutrition and Metabolism
AML	Acute myeloid leukemia
OTU	Operational taxonomic units
LBP	*Lactobacillus plantarum*
IMD	Intestinal mucosal damage
